# A systematic review on the association of birth intervals and risk of autism spectrum and attention deficit hyperactivity disorders

**DOI:** 10.1192/j.eurpsy.2023.1564

**Published:** 2023-07-19

**Authors:** R. Kelishadi

**Affiliations:** Isfahan University of Medical Sciences, Isfahan, Iran, Islamic Republic Of

## Abstract

**Introduction:**

Pregnancy interval may have various impacts on psychiatric and psychologic disorders of the offspring.

**Objectives:**

This systematic review aimed to assess the relationship of short and long inter-pregnancy intervals (IPIs) with the risk of autism spectrum disorder (ASD) and attention deficit hyperactivity disorder (ADHD).

**Methods:**

We performed a systematic search on electronic databases including Pubmed, Web of Science, Scopus, and Embase. We included observational studies that evaluated the association between IPIs and the risk of ASD and ADHD. Two reviewers independently screened and then extracted data on study characteristics, IPIs/ birth intervals, and outcome measures. The methodological quality of the included studies was evaluated following the Joanna Briggs Institute (JBI) critical appraisal checklist.

**Results:**

At the final step, 19 out of 161 studies were included in our systematic review. Among them, 16 and 5 studies assessed the association between IPI and the risk of ASD and ADHD, respectively. In 9 studies, findings supported the association between short intervals and an increased risk of ASD. In addition, 7 studies reported significant association between both short and long intervals and an increased risk of ASD. Moreover, 3 studies demonstrated an association between short intervals and ADHD risk, while long birth interval was merely assessed in 2 studies with conflicting results.

**Conclusions:**

This systematic review strongly confirmed the association of short and long birth intervals with ASD and ADHD. Future studies should investigate the mechanisms underlying these associations and the possible modifiers to decrease the risk of such disorders.

**Disclosure of Interest:**

None Declared

